# Quantitative Evaluation of Skeletal, Dental, and Soft Tissue Changes After Orthognathic Surgery: A Cephalometric and Statistical Analysis

**DOI:** 10.3390/jcm14207336

**Published:** 2025-10-17

**Authors:** Robert-Paul Avrămuț, Andra-Alexandra Stăncioiu, Serban Talpos, Alexandru Cătălin Motofelea, Malina Popa, Camelia Szuhanek

**Affiliations:** 1 Orthodontic Research Center ORTHO-CENTER, Discipline of Orthodontics I, Faculty of Dental Medicine, “Victor Babes” University of Medicine and Pharmacy Timisoara, 9 No., Revolutiei Bv., 300041 Timisoara, Romania; robert.avramut@umft.ro (R.-P.A.); cameliaszuhanek@umft.ro (C.S.); 2 Discipline of Oral and Maxillo-Facial Surgery, Faculty of Dental Medicine, “Victor Babes” University of Medicine and Pharmacy Timisoara, Revolutiei Boulevard 9, 300041 Timisoara, Romania; 3 Center for Molecular Research in Nephrology and Vascular Disease, Discipline of Nephrology, Department VII/Internal Medicine II, Faculty of Medicine, “Victor Babes” University of Medicine and Pharmacy, 300041 Timisoara, Romania; alexandru.motofelea@umft.ro; 4 Department of Pediatric Dentistry, Faculty of Dental Medicine, “Victor Babes” University of Medicine and Pharmacy, Eftimie Murgu Square 2, 300041 Timisoara, Romania; popa.malina@umft.ro

**Keywords:** cephalometric analysis, orthognathic surgery, skeletal discrepancy, facial aesthetics, interdisciplinary treatment

## Abstract

**Background/Objectives:** Combining orthognathic surgery with orthodontic therapy is a crucial approach for correcting severe dentofacial deformities that orthodontics alone cannot address. This study aimed to quantify skeletal, dental, and soft tissue alterations following orthognathic surgery and to assess correlations among cephalometric parameters to improve understanding of treatment outcomes. **Methods:** A prospective observational study was conducted on 25 Romanian patients (44% female and 56% male; median age, 28 years) who underwent orthognathic surgery. Standardized pre- and postoperative lateral cephalometric radiographs were analyzed using WebCeph 2.0.0 software. The evaluated parameters included the SNA angle (sella–nasion–point A, indicating maxillary position), SNB angle (sella–nasion–point B, indicating mandibular position), ANB angle (maxillo-mandibular relationship), Pog-N-Perp (distance from pogonion to the nasion-perpendicular line), U1–NA° (inclination of the upper incisor relative to the maxillary base), L1–NB° (inclination of the lower incisor relative to the mandibular base), nasolabial angle, and facial convexity. Statistical analyses included paired *t*-tests and correlation analysis. **Results:** Significant anterior repositioning of the maxilla was observed, with SNA increasing from 83.6° to 86.3° (*p* = 0.019). The SNB angle remained stable, while ANB increased toward normalized sagittal relationships (0.9° to 3.0°, *p* = 0.060). Soft tissue analysis revealed a slight increase in the nasolabial angle (102° to 105°) and improved facial convexity. Strong correlations were found between skeletal parameters (SNB and ANB, r = −0.852, *p* < 0.001) and between skeletal and dental variables (ANB and L1–NB°, r = 0.652, *p* < 0.001), confirming coordinated skeletal–soft tissue adaptation. **Conclusions:** Orthognathic surgery significantly enhances skeletal balance and facial harmony, particularly through maxillary advancement. The integration of virtual surgical planning and interdisciplinary collaboration improves accuracy, predictability, and patient-centered outcomes in surgical orthodontics.

## 1. Introduction

Orthognathic surgery involves the surgical treatment of mandible, maxilla, or both skeletal defects. The fundamental defect might be present at birth and is known as congenital or intrinsic. Dental compensations related to the malocclusion and the underlying skeletal deformity must be taken into account when measuring these discrepancies because of the limitations of non-surgical therapies for correcting them and the relationship between facial skeletal deformities and masticatory dysfunction.

Patients were indicated for orthognathic surgery based on clinically significant skeletal discrepancies exceeding two standard deviations from normal values. Typical criteria included anteroposterior discrepancies with overjet ≥ +5 mm or negative overjet, molar relationship differences ≥ 4 mm, vertical deformities such as anterior or posterior open bite > 2 mm or deep overbite with soft-tissue impingement, transverse discrepancies ≥ 4 mm (bilateral) or ≥3 mm (unilateral), and facial asymmetries > 3 mm associated with occlusal deviation.

When there are clear indications of malfunction, orthognathic surgery could be the best course of action. Conditions involving airway dysfunction, such as sleep apnea, TMJ problems, psychological issues, and speech difficulties, may fall under this category [1].

Many people have started using 3D computer-assisted technology to help with clinical evaluations and treatment plans since it combines 3D imaging with CAD and manufacturing procedures. To enhance surgical planning and results, this technology has lately been used in orthognathic surgery. To improve surgical accuracy and multidisciplinary collaboration, modern orthognathic procedures are progressively incorporating digital processes. These include intraoperative navigation systems, CAD/CAM-fabricated splints, three-dimensional virtual surgical planning, and cone-beam computed tomography (CBCT) [2].

The primary treatment for patients who are too old for growth modification and for those with dentofacial deformities too severe to be corrected by orthodontic camouflage alone is orthognathic surgery to realign the maxilla, mandible, and chin. Standard orthognathic treatments to repair jaw deformity and adjuvant procedures to improve hard and soft tissue shapes comprise today’s orthognathic surgical treatment for dentofacial deformity. These supplementary operations include neck suction lipectomy, septorhinoplasty, and osseous versus alloplastic genioplasty. To effectively develop and carry out a thorough treatment plan with predictable results, the orthodontist and maxillofacial surgeon must work together [3].

In the realm of craniofacial surgery, orthognathic surgery is frequently used to treat obstructive sleep apnea (OSA), malocclusion, and issues with the facial profile. Repositioning the maxilla, mandible, and chin is the aim of orthognathic surgery. Commonly used techniques include bilateral sagittal split osteotomy (BSSO) with or without osseous genioplasty and LeFort I osteotomy [4].

In 1849, American surgeon Simon P. Hullihen conducted the first mandibular osteotomy to surgically rectify prognathism and class III malocclusion. While there was a clear limitation in the treatment of malocclusion following the surgery, resulting in edge-to-edge malocclusion anteriorly, the skeletal prognathism was rectified postoperatively. In order to address both the skeletal profile and malocclusion, orthodontic treatment has gained popularity since the 1970s. Orthognathic surgery is frequently used in conjunction with orthodontic treatment [4].

When a child or adult is diagnosed with a dentofacial abnormality that may require surgery, it is critical that they have a thorough evaluation by both an orthodontist and a maxillofacial surgeon. The patient is examined, all records are reviewed, and the patient and family are informed of the possible treatment options by the maxillofacial surgeon. Achieving both functional (occlusal) and facial cosmetic goals is the maxillofacial surgeon’s main focus in this conversation. Complete records are obtained by an orthodontist, including dental models, centric bite impressions, lateral and panorex radiographs, and face and occlusal radiographs. After that, the patient is given a recommended treatment plan that has been reviewed and arranged by the surgeon and orthodontist [5].

The primary goal of preoperative orthodontics is to align and level teeth over the basal bone. Correcting (reversing) dental compensation, maintaining the dental midline, and developing appropriate incisor inclination and transverse arch width are a few examples of particular objectives. Dr. John Wirthlin provides an authoritative review of these factors. Mandibular or maxillary surgery, or both, may be part of the orthognathic surgical treatments. To enhance nasal airflow dynamics, concurrent intranasal surgery involving septoplasty and inferior turbinate reduction might be necessary. To enhance the overall aesthetic result, certain patients may also be candidates for genioplasty and neck liposuction. Four to six weeks following the procedure, postoperative orthodontic treatment often begins. A postorthodontic retention phase starts after the orthodontist completes the final occlusion details [3].

Both the orthodontist and the treating maxillofacial surgeon should carefully consider psychological aspects. The underlying reasons for seeking therapy for skeletal jaw deformity, the condition’s psychosocial impact, and the patient’s psychosocial reaction to treatment must all be understood by the treating team. Anticipating and aligning the suggested treatment plan with the patient’s expectations is crucial. Counseling the patient on the expected course of rehabilitation, recovery period, common problems, and surgical sequelae is equally crucial. For the first four to six weeks after the procedure, the patient should be made aware of the sudden changes in lifestyle that take place [3].

The collaboration between orthodontists and maxillofacial surgeon in orthognathic surgery originated in St. Louis, Missouri, in the late 19th century. During that period, Vilray Blair, a preeminent orthognathic surgeon, collaborated with Edward Angle, who is regarded as the Father of Modern Orthodontics. In close cooperation, these two executed the first ostectomy of the mandible in a patient with mandibular prognathism in 1898. Despite significant advancements in orthognathic surgery since the early 1900s, the orthodontist’s involvement in attaining optimal outcomes remains essential. Orthognathic surgery may rectify significant aesthetic and functional defects, serving as a transformative experience for the patient. The orthodontist’s involvement in orthognathic surgery encompasses many phases: initial examination, presurgical orthodontics, surgical planning, and postsurgical orthodontics. Collaboration between the orthodontist and the surgeon is essential at each of these stages. The collaboration between the orthodontist and the maxillofacial surgeon during this period is crucial for achieving a satisfactory result [6].

Presurgical orthodontic therapy is often a crucial element of an orthognathic surgery procedure. The main goal is to construct dental arches that occlude correctly after the surgical rectification of the skeletal discrepancy. The degree of incisor decompensation achieved during presurgical orthodontic therapy influences the amount of jaw relocation following surgery and is a critical determinant of treatment success or failure [7,8,9,10].

Orthognathic therapy planning includes a psychological evaluation. Aside from clinical examination, validated tools like the Orthognathic Quality of Life Questionnaire (OQLQ) may objectively examine the social, aesthetic, and functional elements of dentofacial abnormalities, as well as the patient’s motivation and psychosocial adaptability [11].

Orthognathic surgery significantly enhances patients’ physical, mental, and social well-being, according to recent systematic data [12].

Both maxillary and mandibular treatments can result in a wide range of problems, which can have a direct impact on the outcome either early or late after surgery. Nerve damage/sensitivity change, temporomandibular joint (TMJ) issues, hemorrhage, auditory tube dysfunction, infection, bad split, nonunion of segments, skeletal relapse, bone necrosis, soft tissue injuries, positional vertigo, dental complications, postoperative swelling, and psychological depression are the most commonly reported post-surgical complications [13].

To avoid soft-tissue damage and the danger of postoperative sensory disruption, current orthognathic surgery frequently employs preventative techniques such as intraoperative monitoring of the inferior alveolar nerve and piezosurgery for accurate osteotomies [14,15].

Optimizing recovery after surgery often involves pharmacological assistance. New research indicates that if vitamin D insufficiency is detected and treated before mandibular or orthognathic surgery, bone metabolism may be improved and recovery can occur more quickly. Having enough vitamin D in the body speeds up the rebuilding of bone and reduces the likelihood of surgical problems and delayed union [13].

Postoperative problems after orthognathic surgery may be significantly reduced with the use of systemic and pharmaceutical therapy. Patients with metabolic abnormalities, systemic comorbidities, or nutritional deficiencies (such as vitamin D deficiency) are at a higher risk of delayed healing and infection, according to a recent analysis. Optimizing the patient’s systemic state with antibacterial prophylaxis, anti-inflammatory medicine, and nutritional or vitamin supplements can greatly enhance recovery and bone regeneration [16].

The majority of research on the postoperative results related to orthognathic surgery, either surgically or patient-wise, has been conducted on a normal demographic group without taking systemic comorbidities into account [16].

A preventative protocol needs to be prepared beforehand, and patients with systemic comorbidities may present a particular risk profile that a surgeon should be ready to manage should it arise. For example, surgical tissue necrosis and lengthier hospital stays are more likely to occur in patients with diabetes mellitus (DM). Chronic obstructive pulmonary disease (COPD), Crohn’s disease, and vitamin D deficiency all affect bone metabolism, which may lead to increased post-traumatic bone loss and delayed bone recovery. Last but not least, thalassemia patients are more likely to experience bleeding and become infected [16]. In thalassemia patients, multiple organ systems may be affected by the disease, transfusion history, iron overload, and therapy; thus, complication risks depend on disease severity and subtype. In our study, the patient with thalassemia was diagnosed with β-thalassemia minor, a mild form of the disease that typically does not increase the risk of postoperative complications compared with healthy individuals [17].

Thorough preoperative examination and meticulous perioperative treatment are crucial for the safe and predictable outcomes of orthognathic surgery. A comprehensive evaluation, according to the American Association of Oral and Maxillofacial Surgeons’ (AAOMS) Parameters of Care: Clinical Practice Guidelines—Patient Assessment (ParCare 2023), involves determining the patient’s ASA physical status, obtaining and documenting informed consent, and finding any systemic conditions that could impact the risk of surgery. Diabetes patients should undergo glycemic optimization by having their HbA1c levels checked recently, taking measures to prevent hypoglycemia, and making adjustments to their insulin or oral hypoglycemic medication as needed. Patients taking antiresorptive or anti-angiogenic medications should also receive extra care to avoid medication-related osteonecrosis of the jaw (MRONJ). Early patient identification, frequent dental examination, and surgical methods aimed at reducing stress and improving recovery are among the recommendations made by the AAOMS ParCare 2023-Patient Assessment for patients at risk of MRONJ. To lessen the likelihood of problems after surgery, it is crucial to take preventative steps, such as practicing excellent dental hygiene and keeping in close contact with the prescribing doctor. Orthognathic surgery may benefit from a standardized framework that incorporates these structured, evidence-based guidelines to optimize patients and reduce perioperative risk [18].

The ability for bone remodeling and the tissue reaction should be meticulously evaluated during orthognathic surgeries, as shown by a case involving bone augmentation in an acromegalic patient [19].

The aim of this study is to quantitatively evaluate skeletal, dental, and soft tissue changes after orthognathic surgery using standardized cephalometric and statistical analyses. Beyond objective measurement, it highlights the value of interdisciplinary collaboration between orthodontists and maxillofacial surgeons in correcting functional and anatomical imbalances while improving facial aesthetics, occlusion, and patient satisfaction. The study also recognizes the psychosocial benefits of surgery, including enhanced self-esteem and social confidence. By incorporating digital tools and virtual planning from diagnosis to follow-up, it emphasizes precision, predictability, and customization in modern protocols.

## 2. Materials and Methods

The study concentrated on patients enrolled in the Faculty of Dental Medicine at the “Victor Babes” University of Medicine and Pharmacy in Timisoara who were enrolled in the Orthodontics I Discipline. The study was approved by the Institutional Ethics Committee of the “Victor Babes” University of Medicine and Pharmacy in Timisoara, Romania, and each participant provided written informed permission (CECS Nr. 105/02.11.2022 rev 2025).

The study included patients who underwent orthognathic surgery between December 2022 and December 2024, with a minimum postoperative observation period of six months.

At the Faculty of Dental Medicine, “Victor Babes” University of Medicine and Pharmacy, Timisoara, 25 adult patients (14 men and 11 females; median age = 28 years, interquartile range = 24–33 years) from Romania had a combination of orthodontic and orthognathic therapy. All of the patients needed surgical correction of dento-maxillofacial abnormalities to have better facial balance and functional occlusion. This sample was representative of the orthognathic surgery patient group at university-affiliated practices, which consists mostly of young adults.

This research examined cephalometric alterations after orthognathic surgery using a within-subject pre-post methodology. Consistent with general methodological guidelines for masticatory muscle and temporomandibular joint research, which state that a sample size of 25 patients allows for the detection of large effects with around 80% power at an alpha level of 0.05 [20], the achieved sample was deemed adequate. This research aimed to identify significant therapeutic impacts, acknowledging that a larger sample size may be necessary to detect smaller effects.

Twenty-five Romanian patients who went to the university center for orthodontic treatment to improve the look of their faces and teeth and achieve a stable and functional occlusion were part of the study. Radiographs like OPGs, lateral cephalograms, study models, and clinical photographs (intraoral and extraoral) were used to do a full clinical review on every patient.

Inclusion and Exclusion Criteria—Patients were eligible for orthognathic surgery if they met all of the following criteria: they were at least 18 years old at the time of the initial evaluation; presented with a dento-maxillofacial deformity requiring surgical correction, including but not limited to skeletal Class II or Class III malocclusion, vertical maxillary excess or deficiency, or transverse discrepancies not manageable with orthodontic treatment alone; had completed or were scheduled to complete presurgical orthodontic treatment; were in good general health (ASA I or II) with no systemic diseases contraindicating general anesthesia; had complete diagnostic records, including CBCT scans, cephalometric radiographs, intraoral scans, and clinical photographs; and provided informed consent for treatment and follow-up assessments. Patients were excluded if they were under 18 years of age; had craniofacial syndromes or congenital anomalies (e.g., cleft lip/palate, hemifacial microsomia); presented with temporomandibular joint disorders requiring alternative or additional surgical treatment; had poor oral hygiene or active periodontal disease at the time of surgical planning; had severe psychiatric disorders or demonstrated non-compliance that could compromise treatment or follow-up; had incomplete diagnostic records; or refused orthodontic treatment or did not provide full informed consent.

### 2.1. Procedure Methodology

All data entry actions were performed in accordance with the General Data Protection Regulation (GDPR) and the platform’s compliance policies. Cephalometric Image Acquisition and Processing After the patient profile was successfully created within the WebCeph platform, a lateral cephalometric radiograph was uploaded to the dedicated cephalometric analysis module. Initially, the AI Landmark Detection tool was used to automatically identify key anatomical reference points on the radiograph. This was followed by AI digitization, which facilitated the transformation of the image into a format suitable for quantitative analysis. To ensure spatial accuracy, image size calibration was performed using the platform’s integrated calibration tool. A reference scale of 10 mm was defined on the radiograph, resulting in a calculated resolution of 5.491 pixels per millimeter (px/mm). This calibration step is essential to maintain dimensional fidelity across different imaging sources. For the anatomical structures to have the best possible clarity, contrast, and dimensional correctness, the technical imaging settings were chosen with care: picture size 2685 × 2539 px, resolution 292 dpi, and 24-bit depth. In order for the AI system and the examiner performing human verification to accurately identify landmarks, it is crucial to have high pixel density and bit depth. This improves edge definition and grayscale discrimination. Measurement accuracy is maintained between patients and imaging sessions due to adequate spatial resolution, which reduces calibration error when scaling the picture (10 mm reference).

Following automatic landmark detection, the position of selected cephalometric landmarks was manually reviewed and adjusted where necessary to enhance anatomical precision. The final configuration of the landmark set was saved within the platform to complete the preprocessing phase for subsequent analysis. Cephalometric Parameter Extraction Following the confirmation and saving of the landmark positions, the analysis proceeded via the pathway: Analysis → Cephalometric Analysis → WebCeph Analysis, Jarabak–Chart. Within this section of the platform, the software automatically generated a comprehensive set of cephalometric measurements. From these, only the specific parameters predefined in the study’s design were selected and extracted for further statistical analysis. For landmark detection, many experts have used WebCeph. For landmark detection, many experts have used WebCeph. To guarantee anatomical accuracy, two seasoned orthodontists thoroughly examined and rectified all automated landmark detections generated by the WebCeph AI module. With intraclass correlation coefficients (ICCs) generally exceeding 0.90 for skeletal and dental parameters and mean differences below 1° or 0.5 mm, WebCeph has been proven to be reliable and accurate in multiple prior studies that showed excellent agreement with conventional manual tracing [21,22,23,24,25,26]. All cephalometric tracings were reviewed for consistency in this investigation, and pre- and postoperative radiographs followed the same standardized methodology. We achieved high reproducibility and reduced measurement bias by using validated software, expert verification, and automated landmark detection.

Cephalometric analyses were conducted using the WebCeph software platform (AssembleCircle Corp., Pangyoyeok-ro, Bundang-gu, Seongnam-si, Gyeonggi-do, Republic of Korea), a digital tracing system developed and registered with the Korean Intellectual Property Office (Seoul, Republic of Korea). The software’s artificial intelligence technologies have been patented by both the Korean and United States Intellectual Property Offices, reflecting its validated innovation in automated cephalometric landmark detection and analysis.

Mean Values and Standard Deviations from the WebCeph program are:

The SNA angle (sella–nasion–point A, representing maxillary position) averaged 81.77° (SD = 6.0); the SNB angle (sella–nasion–point B, representing mandibular position) averaged 80.42° (SD = 5.3); and the ANB angle (maxillo-mandibular relationship) averaged 2.05° (SD = 1.8). The U1–NA° (upper incisor inclination to the maxillary base) measured 22° (SD = 5.0), and the L1–NB° (lower incisor inclination to the mandibular base) measured 25° (SD = 5.0). The nasolabial angle was 95° (SD = 5.0), the Pog–N-Perp (FH) (distance from pogonion to the nasion-perpendicular line in the Frankfort horizontal plane) was −0.3 mm (SD = 3.8), and facial convexity measured 1.3° (SD = 2.4) based on Jarabak analysis.

SNA angle—The SNA angle measures where the maxilla is in relation to the base of the skull in the sagittal plane. This number should be 82° ± 2°. Higher numbers mean that the maxilla sticks out. Lower numbers mean the maxilla is retracted [27].

SNB angle—The SNB angle measures where the lower jaw is in relation to the base of the skull in the sagittal plane. It should be 80° ± 2°. Higher numbers mean that the mandible sticks out. Lower numbers mean the mandible is retrusive [27].

ANB angle—In the sagittal line, the ANB angle measures how far apart the mandible and maxilla are from each other. It is normal for it to be 2 ± 2°. Skeletal class II pattern for higher values. Skeletal class III pattern refers to numbers that are low or even negative [25].

U1–NA ° (deg)—This angle is made up of the maxillary central incisors’ long axis (the edge of the tooth to the root’s tip) and the NA plane.

L1–NB ° (deg)—There is an angle of this size between the NB plane and the long line of the upper front teeth.

In skeletal class I, Mathapun, J et al. found U1-NA (degree) 23.25 ± 4.07 and L1-NB (degree) 32.03 ± 3.43 [28].

Nasolabial angle—The nasolabial angle is the shape made by the line meeting the columella and the upper lip. The normal number is 102°. Higher numbers = lips that stick out. Lower numbers mean lips that stick out [27].

Facial convexity—It is made up of the line N-A and the line A to Pg. It is also known as the angle of face convexity. Measuring the length and width of a skeleton: N-A-Pg angle (degree of skeletal convexity), with N-A-Pg (°); mean 3.9 S.D. 6.4 [29].

Pog to N-Perp (FH) (mm)—there is a distance between the Pogonion point and N-perpendicular. Pog to N-perp-ASO Group—Mean 10.41, S.D. 6.80; Non-ASO Group Mean −8.84 S.D. 5.94 [30].

For their excellent diagnostic utility in assessing skeletal, dental, and soft tissue changes after orthognathic surgery, the cephalometric variables examined in this research (SNA, SNB, ANB, U1-NA, L1-NB, nasolabial angle, facial convexity, and Pog-N-Perp) were chosen. The anteroposterior relationships of the maxilla and mandible (SNA, SNB, ANB), the incisor inclination and dental compensation (U1-NA, L1-NB), and the harmony of the face profile and soft tissues (nasolabial angle, facial convexity, Pog-N-Perp) are all accurately represented by these parameters. We prefer these metrics over more comprehensive systems like Steiner, McNamara, or Tweed due to their clinical usefulness and widespread acceptance in evaluating surgical outcomes. These systems contain variables that are less surgery-sensitive or have overlapping components.

As a point of reference, lateral cephalograms of every patient were taken with their heads in their natural postures. Patients were positioned with their heads straight over the chin rest, shoulders relaxed, back straight, feet closed, tongue against the hard palate, and the Frankfort plane parallel to the floor and the median sagittal plane perpendicular to the ground during radiography exposure. For cephalometric radiographs, patients were placed in a natural head position, with the gaze straight ahead, the lips in relaxed contact, and the teeth in central occlusion. Under gentle pressure from the ear rods of the cephalostat, the patients were positioned with the external auditory meatus and the Frankfort horizontal plane parallel to the floor. The quality and sharpness of the radiography images were evaluated [24].

Pre- and post-operative lateral cephalometric radiographs were all taken with the same exposure settings, including focal distance, tube voltage, and current, and from the same radiography device. All image acquisitions were carried out by the same qualified radiology technician in accordance with a predetermined methodology to ensure consistency from session to session. To guarantee consistent alignment, patients were placed in the normal head posture with their teeth in centric occlusion and the Frankfort horizontal plane parallel to the floor. The built-in cephalostat was used to verify this alignment. The reliability of inter-timepoint cephalometric comparisons was enhanced by this stringent uniformity, which eliminated positional and exposure variability.

Qualified radiography technicians obtained lateral cephalometric radiographs following conventional clinical imaging procedures. The PaX-i3D Green imaging system (Vatech Co., Ltd., Hwaseong, Republic of Korea; manufactured in 2019), a hybrid digital cephalometric device renowned for its low radiation dose and high-resolution image quality, was used to acquire all radiographs. Standard exposure parameters for lateral cephalograms on the PaX-i3D Green system were as follows: Tube voltage: 70–90 kVp, tube current: 5–10 mA, exposure time: approximately 1.0–1.5 s, and image resolution: 0.2–0.3 mm voxel size (depending on patient size and mode selected). All images were saved in JPG (.jpg) format and subsequently uploaded to the WebCeph cloud-based platform for digital tracing, landmark identification, and cephalometric analysis.

This study was conducted on patients presenting with complex dentomaxillary deformities requiring orthognathic surgical correction. A standardized, interdisciplinary workflow was implemented, consisting of the following sequential phases:

1. Initial Clinical Consultation—The first phase involved a multidisciplinary consultation between the patient, oral and maxillofacial surgeons, and orthodontists. Clinical findings were reviewed, and the patient was informed about the therapeutic process, including objectives, stages, risks, and expected outcomes.

2. Diagnostic Data Acquisition—Comprehensive diagnostic data were collected, including extraoral and intraoral clinical photographs, digital or conventional dental impressions (or intraoral scans), panoramic radiographs (orthopantomograms), lateral cephalograms, and cone-beam computed tomography (CBCT) scans. These records formed the basis for diagnosis and virtual treatment planning.

3. Interdisciplinary Evaluation and Treatment Planning—A detailed case review was performed to establish the diagnosis, treatment objectives, and surgical–orthodontic plan. A financial estimate and overall treatment timeline were also communicated to the patient.

4. Orthodontic Preparation—For patients who had not previously undergone orthodontic therapy, treatment was initiated to achieve dental decompensation. This orthodontic phase aligned the dentition in preparation for skeletal repositioning and followed standard protocols for presurgical orthodontics in orthognathic cases.

5. Pre-Surgical Records and 3D Planning—Upon completion of the orthodontic phase, updated diagnostic records were obtained, including a full-head CBCT scan and new intraoral scans. These data were used for three-dimensional (3D) virtual surgical planning to ensure precise skeletal movements.

6. Virtual Surgical Planning and Splint Fabrication—Digital planning was performed using NEMO-FAB^®^ software V25 (Software Nemotec, Madrid, Spain). Virtual osteotomies and maxillomandibular repositioning were simulated to determine the final surgical outcome. Based on the digital plan, surgical splints (initial, intermediate, and final) were fabricated using CAD/CAM technology.

7. Splint Verification and Surgical Scheduling—The accuracy and fit of the surgical splints were verified clinically. Following approval of the final splint set, the surgical procedure was scheduled.

8. Surgical Procedure—Orthognathic surgery was performed under general anesthesia via rhino-tracheal intubation. Prefabricated surgical splints guided intraoperative maxillofacial repositioning according to the virtual plan.

9. Postoperative Management and Orthodontic Finalization—Postoperative evaluations were performed to monitor healing, skeletal stability, and functional recovery. The final orthodontic phase refined occlusion and optimized both aesthetic and functional outcomes.

The cephalometric parameters analyzed include SNA (anteroposterior position of the maxilla), SNB (anteroposterior position of the mandible), ANB (maxillomandibular discrepancy), U1 to NA (°) (inclination of upper incisors relative to the maxilla), L1 to NB (°) (inclination of lower incisors relative to the mandible), Nasolabial angle (soft tissue profile), Facial convexity angle, and Pogonion to Nasion-Perpendicular (Pog to N-Perp, relative to the Frankfort horizontal plane). By comparing preoperative and postoperative cephalometric values, the study aims to objectively assess the impact of orthognathic surgery on facial esthetics and skeletal balance.

**Figure 1:**
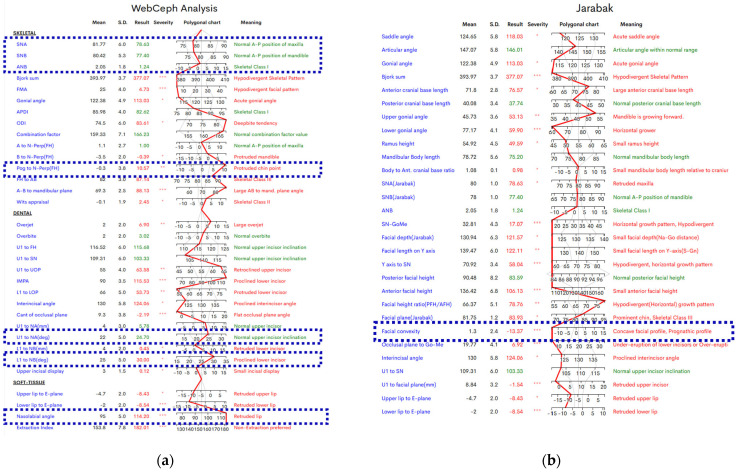
Cephalometric Analysis (Pre-Surgical Values): (**a**) WebCeph Standard; (**b**) Jarabak. Asterisks indicate the severity of deviation from normal values: * = mild, ** = moderate, *** = severe. Absence of an as-terisk denotes values within normal limits.

Figure 1a,b illustrate the cephalometric analysis performed using WebCeph, a digital platform for assessing skeletal, dental, and soft tissue relationships. The figures represent the pre-orthognathic surgery values for the patient included in the study. Two distinct methods are illustrative—(a) WebCeph Analysis and (b) Jarabak Analysis—and all parameters used in this study are enclosed in dark blue dashed boxes in both figures and represent the patient’s cephalometric values prior to orthognathic surgery. These measurements were selected for their diagnostic relevance and will serve as a baseline for post-treatment comparison.

**Figure 2:**
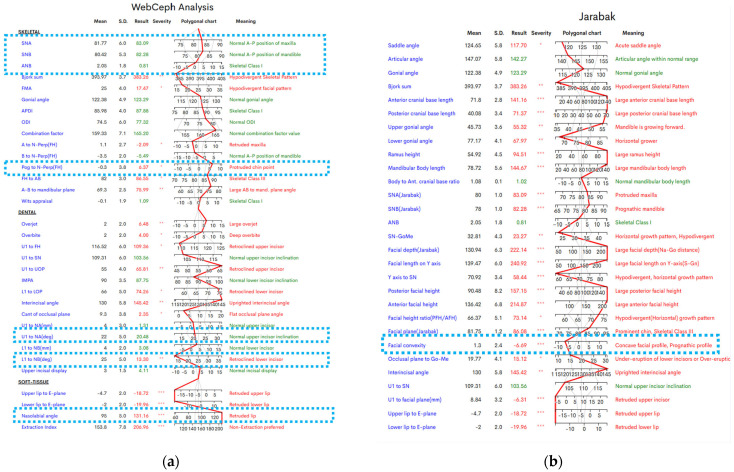
Cephalometric Analysis (Post-Surgical Values): (**a**) WebCeph Standard; (**b**) Jarabak. Asterisks indicate the **severity of deviation from normal values**: * = mild, ** = moderate, *** = severe. Absence of an asterisk denotes values within normal limits.

Figure 2 illustrates the post-orthognathic surgery cephalometric analysis performed using WebCeph, a digital platform designed to assess skeletal, dental, and soft tissue relationships. The figures represent the post-treatment values for the same patient previously evaluated before surgery. Two distinct analysis methods are shown: (a) WebCeph Standard Analysis and (b) Jarabak Analysis.

In both figures, the parameters used in this study are highlighted with light blue dashed boxes, allowing direct identification of the specific measurements selected for evaluation. These values reflect the skeletal and soft tissue changes achieved following surgical correction and orthodontic treatment. The post-operative measurements are used to assess treatment success and compare outcomes against the pre-treatment baseline.

**Figure 3:**
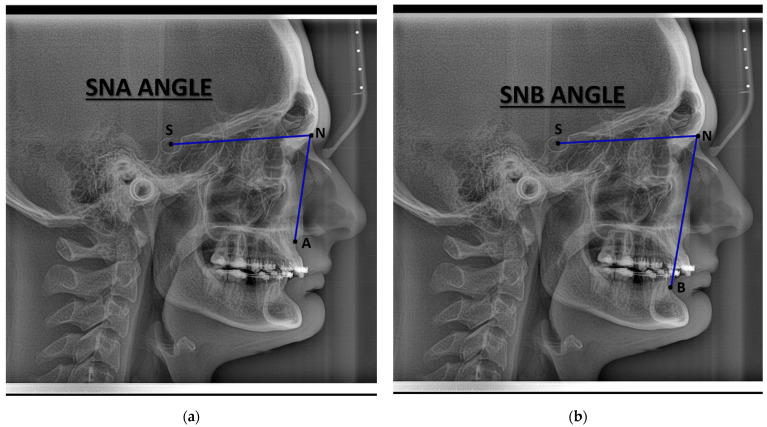
(**a**) Cephalometric Representation of SNA Angle and (**b**) Cephalometric Representation of SNB Angle.

Figure 3a: Lateral cephalometric radiograph illustrating the SNA angle, used to assess the anteroposterior position of the maxilla relative to the cranial base. This angle is formed by the intersection of lines drawn from Sella (S) to Nasion (N) and from Nasion (N) to Point A, which represents the deepest midline point on the premaxilla between the anterior nasal spine and the alveolar crest. Figure 3b: Lateral cephalometric radiograph depicting the SNB angle, which evaluates the anteroposterior position of the mandible in relation to the cranial base. The angle is formed by lines connecting Sella (S) to Nasion (N) and Nasion (N) to Point B, the most posterior point on the outer contour of the mandibular alveolar process. The patient shown is in the pre-surgical phase of orthognathic treatment, and both SNA and SNB angles were used as parameters in the present study to assess skeletal jaw relationships.

**Figure 4:**
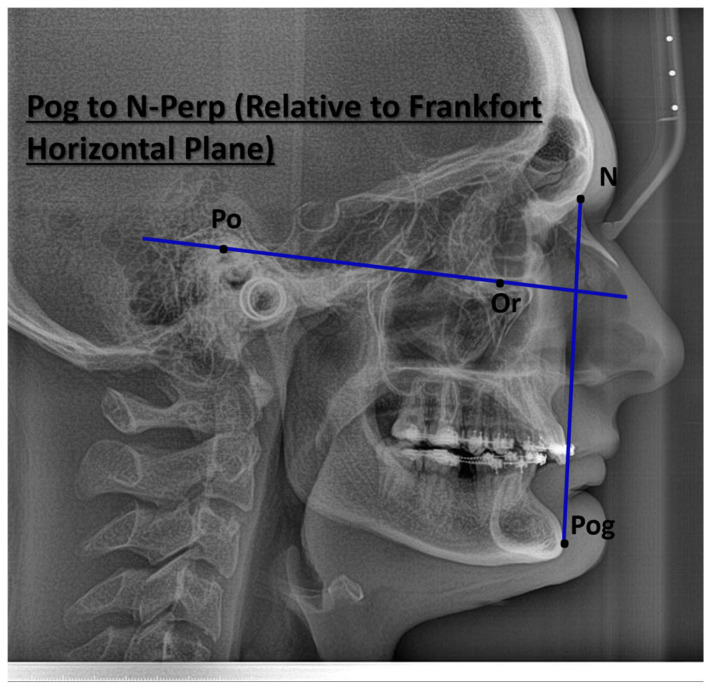
Cephalometric Assessment of Pogonion Position Relative to Nasion Perpendicular and Frankfort Horizontal Plane.

Figure 4 illustrates the measurement of the distance from Pogonion (Pog) to the Nasion Perpendicular (N-Perp) line, referenced relative to the Frankfort Horizontal Plane (FHP). This parameter is fundamental in cephalometric analysis for assessing the anteroposterior position of the mandible. The Pog-N-Perp measurement provides critical diagnostic information for identifying skeletal Class II or Class III discrepancies, aids in the formulation of orthodontic and orthognathic treatment plans, and contributes to the evaluation of facial esthetics and sagittal facial balance in profile analysis.

**Figure 5:**
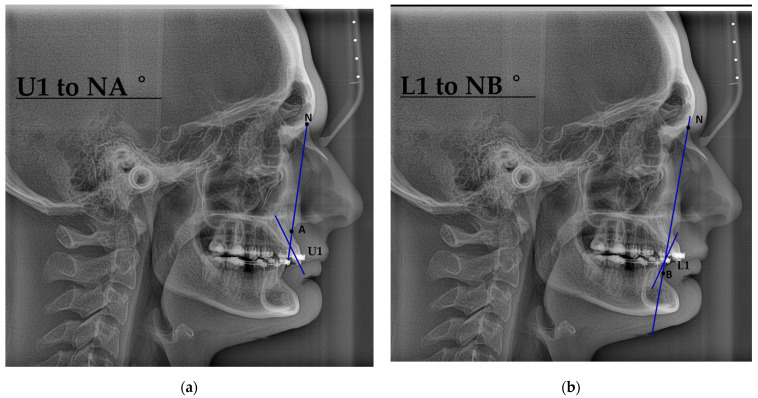
Cephalometric analysis: (**a**) U1 to NA angle (maxillary incisor inclination), (**b**) L1 to NB angle (mandibular incisor inclination).

Figure 5 presents lateral cephalometric radiographs showing incisor inclination relative to skeletal landmarks. (a) U1 to NA angle: The angle between the long axis of the maxillary central incisor (U1) and the line connecting Nasion (N) to Point A. This measurement evaluates the anteroposterior position and inclination of the upper incisors. (b) L1 to NB angle: The angle between the long axis of the mandibular central incisor (L1) and the line connecting Nasion (N) to Point B. This is used to assess the position and angulation of the lower incisors in relation to the mandibular skeletal base. These measurements are crucial for orthodontic and orthognathic diagnosis, treatment planning, and outcome evaluation, particularly in cases involving skeletal malocclusion.

**Figure 6:**
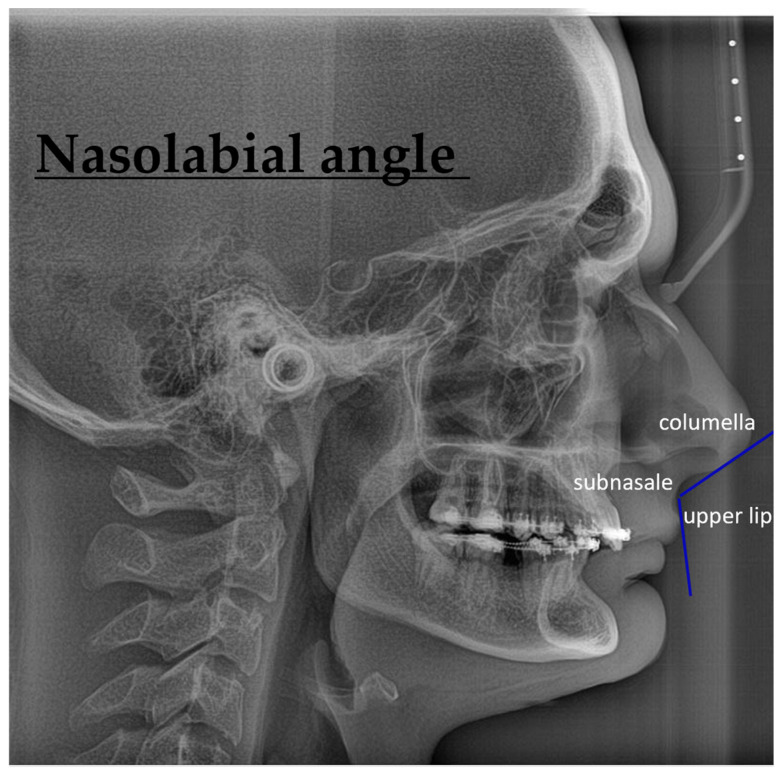
Lateral cephalometric radiograph showing the nasolabial angle, formed by the columella, subnasale, and upper lip.

Figure 6 is a lateral cephalometric radiograph illustrating the nasolabial angle, an important soft tissue parameter in facial esthetics and orthodontic/surgical planning. The angle is formed between the columella (the underside of the nose), the subnasale (junction between the nose and upper lip), and the upper lip. This angle reflects the relationship between the nose and upper lip and is often evaluated before and after orthognathic or rhinoplasty procedures to assess aesthetic balance and soft tissue changes.

**Figure 7:**
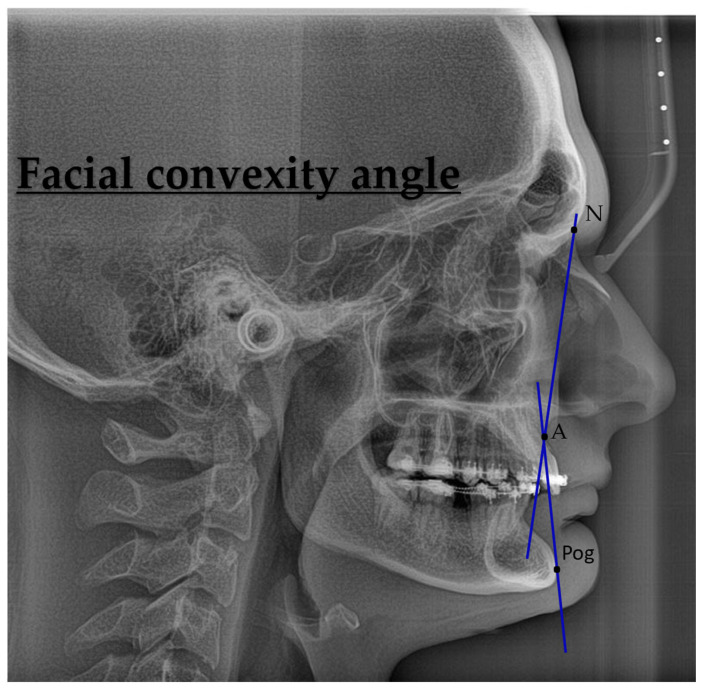
Facial convexity angle (N–A–Pog).

Figure 7 shows lateral cephalometric radiographic measurements used to assess facial skeletal harmony and soft tissue profile. (a) Facial convexity angle: Formed by three skeletal landmarks—Nasion (N), Point A (A), and Pogonion (Pog). This angle reflects the sagittal profile convexity of the face and is useful in evaluating maxillomandibular relationships and facial aesthetics.

**Figure 8:**
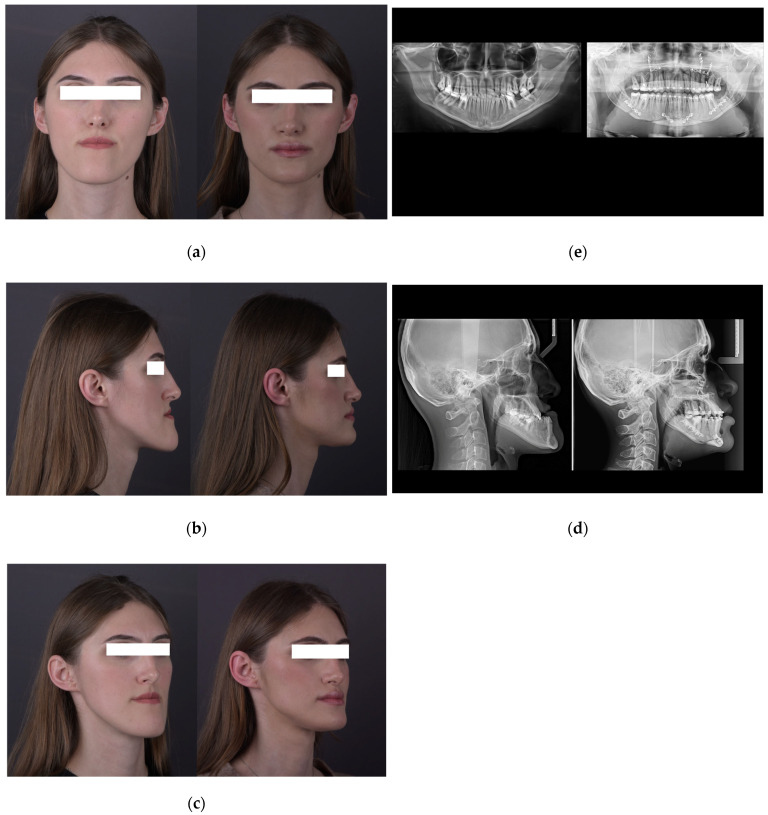
(**a**,**e**) Frontal facial view and panoramic X-rays, pre- and post-surgery: Improved midface projection, facial symmetry, and postoperative bone fixation. (**b**,**d**) Lateral profile and lateral cephalometric X-rays: Balanced facial profile after maxillary advancement and mandibular retrusion; fixation visible postoperatively. (**c**) Semi-profile view: Enhanced jawline contour and facial proportions following surgery.

In Figure 8, we present preoperative (left) and postoperative (right) documentation of a female patient treated with combined orthognathic surgery, including maxillary advancement with a three-piece segmental Le Fort I osteotomy, mandibular setback (bilateral sagittal split osteotomy), and chin reduction genioplasty. The figure illustrates corresponding clinical and radiographic views, grouped to demonstrate the relationship between soft-tissue and skeletal changes: (Figure 8a,e) frontal facial view with panoramic radiographs, (Figure 8b,d) lateral facial profile with lateral cephalometric radiographs, and (Figure 8c) semi-profile view showing overall facial balance and symmetry.

(Figure 8a,e) The frontal facial view and corresponding panoramic radiographs show pre- and postoperative improvements, including enhanced midface projection, reduced lower facial height, improved facial symmetry, and clear evidence of postoperative osseous fixation following segmental maxillary osteotomy and mandibular repositioning.

(Figure 8b,d) The lateral facial profile and corresponding lateral cephalometric radiographs demonstrate substantial advancement of the midface and mandibular retrusion, resulting in a more balanced and harmonious facial profile. Postoperative skeletal movements and fixation hardware are visible.

(Figure 8c) The semi-profile facial view confirms the improved contour of the jawline and proportional facial thirds following surgical intervention.

**Figure 9:**
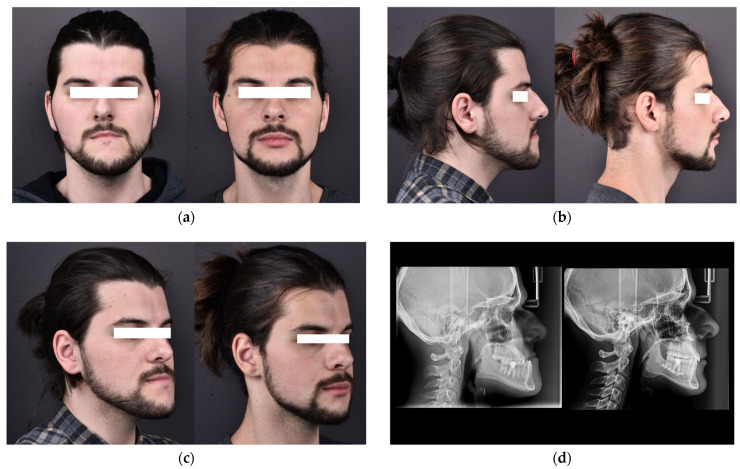
Pre- and postoperative views of the patient after orthognathic surgery (maxillomandibular advancement, mandibular setback, and counterclockwise rotation). (**a**) Frontal view. (**b**) Right profile. (**c**) Left semi-profile. (**d**) Lateral cephalometric radiograph.

Figure 9 shows pre- and postoperative clinical photographs and radiographs of the patient who underwent orthognathic surgery involving maxillomandibular complex advancement, mandibular setback, and counterclockwise rotation.

(9a) Frontal view before (left) and after (right) surgery.

(9b) Right profile view before (left) and after (right) surgery.

(9c) Left semi-profile view before (left) and after (right) surgery.

(9d) Lateral cephalometric radiograph before (left) and after (right) surgery, illustrating skeletal changes and improved jaw alignment.

The patient underwent a combined maxillomandibular advancement with counterclockwise rotation of the occlusal plane, resulting in enhanced facial aesthetics and a more balanced skeletal profile. Mandibular setback addressed the Class III malocclusion, while maxillary advancement improved midfacial projection and occlusal harmony.

### 2.2. Statistical Analysis

All analyses were performed with RStudio v2024.04.2+764 with R v4.4.1. The study used a within-subject pre–post framework in which each participant contributed paired measurements extracted from standardized lateral cephalograms. The primary endpoint was the change in SNA (degrees). Secondary endpoints were the changes in SNB, ANB, Pogonion-to–Nasion-Perpendicular relative to the Frankfort horizontal (mm), U1–NA (degrees), L1–NB (degrees), nasolabial angle (degrees), and facial convexity (degrees).

Continuous variables were summarized as mean ± SD when distributions appeared approximately normal and as median with interquartile range otherwise; categorical variables were summarized as counts and percentages. For each endpoint, the distribution of paired differences (post minus pre) was inspected with histograms and Q–Q plots and formally assessed using the Shapiro–Wilk test. When the paired differences were approximately normal, two-sided paired *t*-tests were used; when normality was untenable, Wilcoxon signed-rank tests were applied. Results are presented as mean (or median) change with 95% confidence intervals, exact *p*-values, and effect sizes. For parametric tests, the effect size was expressed as Cohen’s d, calculated by dividing the mean paired difference by its standard deviation, and accompanied by 95% confidence intervals. In parametric testing, the effect size was represented as Cohen’s d, computed by dividing the mean paired difference by its standard deviation, and accompanied by 95% confidence intervals (CIs). Effect sizes were assessed based on the empirical criteria established by Zieliński and Gawda [20], with values around 0.1, 0.3, and 0.7 denoting small, medium, and large impacts, respectively. The analysis of confidence intervals adhered to the practical guidelines established by O’Brien and Yi [31], highlighting their function in conveying the accuracy and uncertainty of estimations. Furthermore, confidence intervals for correlation coefficients (r) were computed using Fisher’s z-transformation and presented at a 95% confidence level to indicate the dependability of the observed relationships [20,31].

For Wilcoxon tests, effect size r was computed as Z divided by the square root of the sample size, with percentile bootstrap confidence intervals. Multiplicity for secondary endpoints was addressed with Benjamini–Hochberg false discovery rate control at 5%; unadjusted *p*-values and FDR-adjusted q-values are both reported, while the primary endpoint was tested at α = 0.05 without adjustment.

Pre- and post-operative correlation structures were examined using Pearson’s r when bivariate normality and linearity were reasonable; when not, Spearman’s ρ was used as a sensitivity analysis. *p*-values in correlation matrices were FDR-adjusted. If duplicate tracings were available, intra- and inter-examiner reliability were quantified using two-way random-effects intraclass correlation coefficients ICC (2,1) with 95% confidence intervals, method error was calculated using the Dahlberg formula, and Bland–Altman plots were inspected for bias and limits of agreement.

## 3. Results

The study included 25 patients with a median age of 28 years (IQR 24–33), comprising 11 females (44%) and 14 males (56%). This reflects a young adult population, which is typical for individuals undergoing orthognathic surgery.

Analysis of skeletal parameters demonstrated a forward repositioning of the maxilla, as indicated by an increase in the SNA angle from 83.6° (79.9–84.9) preoperatively to 86.3° (80.4–89.0) postoperatively. The SNB angle remained relatively stable, changing only from 83.5° (76.8–87.2) to 84.0° (81.0–86.0), suggesting minimal alteration in mandibular sagittal position. The ANB angle increased from 0.9° (−4.9–5.3) to 3.0° (−0.2–4.5), indicating an improvement toward a more normalized sagittal skeletal relationship. Pogonion to N-perpendicular showed a slight decrease from 1.87 mm (−0.94–2.31) to 1.4 mm (0.4–2.6), reflecting minor adjustments in chin projection relative to the cranial base.

Dental changes were also observed. The maxillary incisors showed retroclination, with the U1–NA angle decreasing from 26° (21–28) to 22° (19–29). Conversely, the L1–NB angle increased slightly from 24° (17–27) to 25° (19–30), indicating a minor proclination of the mandibular incisors. These findings are consistent with the expected dental decompensation following orthognathic surgical correction.

Soft tissue parameters demonstrated favorable esthetic changes. The nasolabial angle increased from 102° (92–114) to 105° (91–117), suggesting a reduction in upper lip protrusion. Facial convexity improved modestly, shifting from 0° (−12–7) pre-operatively to 2° (−6–5) post-operatively, thereby contributing to a more balanced facial profile (Table 1).

The paired samples *t*-test demonstrated a statistically significant increase in the SNA angle following orthognathic surgery (mean difference −3.12°, SE 1.24, *t*(24) = −2.51, *p* = 0.019), indicating a forward advancement of the maxilla. In contrast, the change in SNB was not significant (mean difference −1.15°, *p* = 0.426), suggesting that mandibular sagittal position remained largely unaltered.

The ANB angle showed an increase of 1.98° (SE 1.00), approaching statistical significance (*t*(24) = −1.98, *p* = 0.060), reflecting a trend toward improved sagittal maxillo-mandibular relationship, although the difference did not reach conventional significance. Similarly, the pogonion to N-perpendicular decreased by 1.57 mm (*p* = 0.207), but this was not statistically significant.

Dental parameters revealed no significant differences postoperatively. The U1–NA angle remained stable (mean change 0.47°, *p* = 0.806), while the L1–NB angle showed a tendency toward reduction (mean change −2.97°, *p* = 0.067), approaching but not reaching statistical significance.

Soft tissue changes were modest and not statistically significant. The nasolabial angle increased by an average of 6.95° (*p* = 0.439), and facial convexity improved slightly (mean change −0.50°, *p* = 0.904), but neither reached significance (Table 2).

Since all of the variables were assumed to be approximately normal and linear, we ran a Pearson’s correlation analysis on the whole 25-patient sample (*n* = 25) to see how well the variables fit together. As a sensitivity check, we calculated Spearman’s rank correlation (ρ) when these assumptions were not entirely met, and we found similar tendencies. There were no one-tailed associations. The Benjamini–Hochberg false discovery rate (FDR) correction was used at a 5% level to account for multiple comparisons across variables. To evaluate the accuracy of the estimations, 95% confidence intervals were computed for every correlation coefficient.

**Figure 10:**
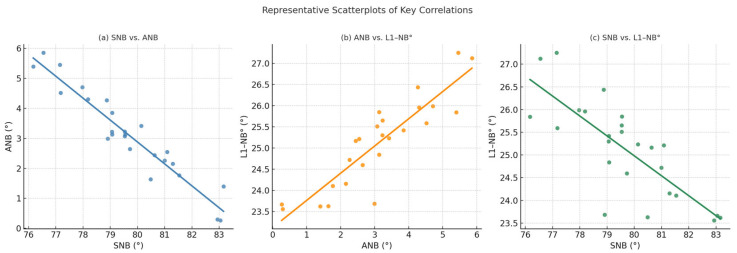
Representative scatterplots illustrating key correlations among cephalometric variables: (**a**) SNB vs. ANB (negative correlation), (**b**) ANB vs. L1–NB° (positive correlation), and (**c**) SNB vs. L1–NB° (negative correlation).

A strong negative correlation was observed between SNB and ANB (r = −0.852, 95% CI: −0.933 to −0.689, *p* < 0.001), indicating that greater mandibular projection was consistently linked with reduced maxillo-mandibular discrepancy. This relationship was complemented by a significant positive correlation between ANB and L1-NB° (r = 0.652, 95% CI: 0.346 to 0.833, *p* < 0.001), highlighting the tendency for greater ANB values to be accompanied by increased lower incisor proclination. Conversely, SNB correlated negatively with L1-NB° (r = −0.559, 95% CI: −0.781 to −0.210, *p* = 0.004), again suggesting the influence of mandibular position on incisor angulation. Facial convexity demonstrated significant associations with both SNB (r = −0.406, 95% CI: −0.691 to −0.013, *p* = 0.044) and ANB (r = 0.432, 95% CI: 0.045 to 0.707, *p* = 0.031), supporting its reliability as an external indicator of skeletal relationships. Several other correlations, including those between age and skeletal or dental parameters, failed to reach statistical significance, underscoring the limited effect of chronological age on preoperative cephalometric measurements in this sample (Table 3, Figure 10).

Figure 10 provides representative scatterplots illustrating the principal correlations among cephalometric parameters in the preoperative dataset.

**Figure 11:**
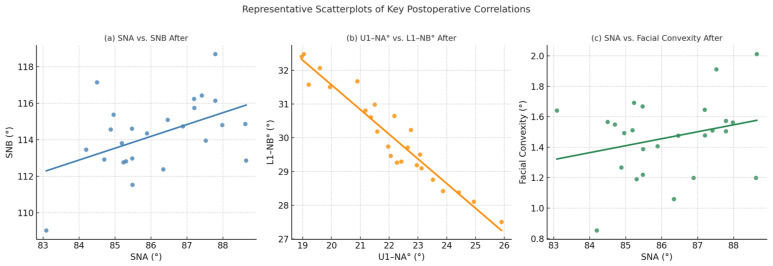
Representative Scatterplots of Key Postoperative Correlations among Cephalometric, Dental, and Soft-Tissue Variables.

Postoperatively, significant associations persisted, and, in some cases, new correlations emerged. SNA-post-operative values were positively correlated with SNB after surgery (r = 0.410, 95% CI: 0.018 to 0.693, *p* = 0.042), suggesting a coordinated change in both maxillary and mandibular positions. ANB after surgery correlated negatively with both SNB after surgery (r = −0.459, 95% CI: −0.723 to −0.078, *p* = 0.021) and Pog to N-Perpendicular (r = −0.425, 95% CI: −0.702 to −0.036, *p* = 0.034), while maintaining a positive association with nasolabial angle (r = 0.435, 95% CI: 0.049 to 0.709, *p* = 0.030). These findings demonstrate that correction of the anteroposterior discrepancy was strongly linked with mandibular advancement and improved soft tissue balance. Of particular note, U1-NA° after surgery exhibited a strong inverse correlation with L1-NB° after surgery (r = −0.696, 95% CI: −0.856 to −0.415, *p* < 0.001), reflecting the compensatory relationship between maxillary and mandibular incisor inclinations during occlusal re-establishment. Facial convexity after surgery also demonstrated significant correlations, including positive associations with L1-NB° (r = 0.420, 95% CI: 0.029 to 0.699, *p* = 0.037) and SNA (r = 0.621, 95% CI: 0.299 to 0.816, *p* < 0.001), and a negative association with U1-NA° after surgery (r = −0.688, 95% CI: −0.852 to −0.403, *p* < 0.001). These results underscore the role of both skeletal and dental changes in determining soft tissue outcomes. Taken together, the correlation analysis revealed a coherent pattern in which mandibular advancement and reduced ANB values were associated with favorable skeletal and dental relationships, as well as improvements in soft tissue profile. Non-significant correlations, such as those between age and cephalometric parameters or between nasolabial angle after surgery and other measurements, further emphasize that the observed changes were predominantly structural and treatment-related rather than age-dependent. Overall, these findings support the stability and predictability of orthognathic surgical corrections, with consistent alignment between skeletal repositioning, dental compensation, and soft tissue harmony in this sample of 25 patients (100%) (Table 4, Figure 11).

**Figure 12:**
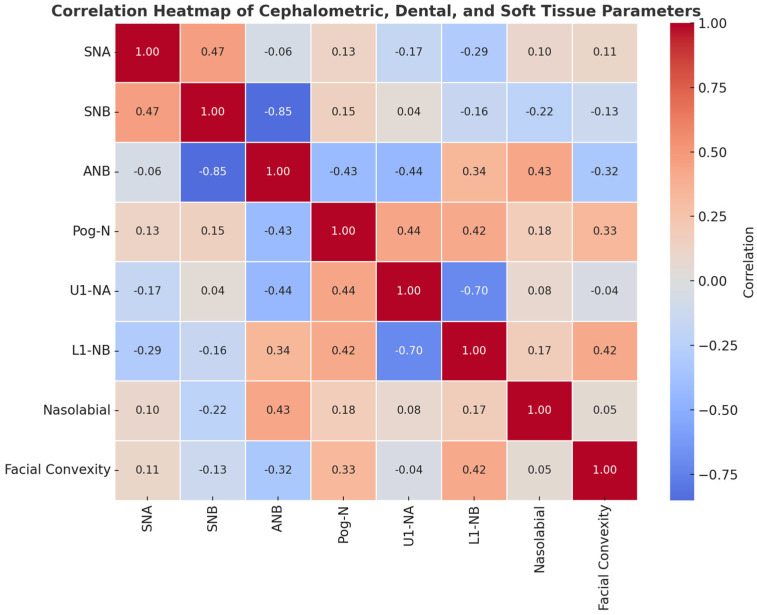
Correlation Heatmap of Cephalometric, Dental, and Soft Tissue Parameters.

Figure 12: Correlation heatmap of cephalometric, dental, and soft tissue parameters. The heatmap illustrates the strength and direction of correlations among key parameters, with red shades indicating positive correlations and blue shades indicating negative correlations. Strong positive correlations were observed between SNA and SNB (r = 0.47) and between Pog-N and L1-NB (r = 0.42), while strong negative correlations were found between SNB and ANB (r = −0.85) and between U1-NA and L1-NB (r = −0.70). Weak or negligible correlations were noted between parameters such as Facial Convexity and U1-NA (r = −0.04) and Nasolabial and Facial Convexity (r = 0.05).

## 4. Discussion

This study evaluated the skeletal, dental, and soft tissue changes following orthognathic surgery in a cohort of 25 patients using standardized digital cephalometric analysis. The results confirm that orthognathic surgery, when combined with comprehensive orthodontic treatment, significantly enhances facial balance and occlusal harmony, with particularly notable effects from maxillary advancement procedures.

Principal Findings: In this study, SNA increased significantly (*p* = 0.019), reflecting anterior movement of the maxilla, while SNB remained stable. ANB increased toward a normalized sagittal relationship (*p* = 0.060). Soft tissue parameters such as the nasolabial angle showed modest, non-significant changes. These findings suggest that skeletal improvements were mainly due to maxillary advancement. These results are consistent with previous studies by Lai et al. (2020) and Huang et al. (2021), which also demonstrated that maxillary advancement is the primary contributor to sagittal correction and improved midface projection [32,33].

Although soft tissue alterations post-orthognathic surgery are often characterized as “subtle yet predictable,” significant interindividual heterogeneity is seen in the response of these tissues to skeletal displacement. Postoperative adaptation is affected by several biological parameters, such as age, sex, soft tissue thickness, and face shape. Lai et al. (2020) showed by three-dimensional imaging that maxillary advancement and rotation substantially influence anteromedial cheek soft tissue displacement, exhibiting a robust linear correlation between bony and soft tissue motions [32].

Huang et al. (2021) similarly discovered that face soft tissue thickness and symmetry significantly vary among patients with mandibular asymmetry and that postoperative soft tissue adaptation strongly correlates with skeletal displacement patterns [33].

These data underscore that the prediction of soft tissue response is relative rather than absolute, contingent upon individual anatomical and physiological characteristics, despite persistent overall tendencies across individuals.

The most significant skeletal change observed was the forward repositioning of the maxilla, evidenced by the statistically significant increase in the SNA angle (*p* = 0.019). This finding demonstrates that maxillary advancement is a key contributor to improved facial projection and midfacial harmony, particularly in Class III skeletal patterns. Conversely, the SNB angle showed minimal changes, indicating that mandibular repositioning was either minor or primarily rotational, preserving mandibular stability postoperatively. The ANB angle increased toward normalized sagittal relationships, reflecting improved maxillo-mandibular balance and confirming the coordinated surgical and orthodontic correction achieved.

Dental parameter changes were modest, with a slight retroclination of the upper incisors and mild proclination of the lower incisors, neither of which reached statistical significance. These results highlight the importance of precise orthodontic preparation and postoperative adjustments to achieve optimal occlusion and smile esthetics.

Soft tissue changes, including a modest increase in the nasolabial angle and improved facial convexity, support the predictable but often subtle soft tissue adaptations following skeletal modifications. These results align with reports by Psomiadis et al. (2025), who emphasized that while skeletal repositioning drives noticeable profile enhancements, soft tissue changes tend to be more conservative due to variable tissue elasticity and thickness [34].

Correlation analysis revealed strong interrelationships between skeletal, dental, and soft tissue parameters. The strong negative correlation between SNB and ANB (r = −0.852, *p* < 0.001) reinforces the role of mandibular positioning in determining sagittal discrepancies, while the positive correlation between ANB and L1–NB° (r = 0.652, *p* < 0.001) demonstrates the compensatory relationship between skeletal base position and incisor inclination. Furthermore, the significant association between facial convexity and both skeletal and dental parameters underscores the clinical relevance of soft tissue evaluation during diagnosis and treatment planning.

From a clinical standpoint, these findings highlight the importance of interdisciplinary collaboration and the integration of digital tools such as WebCeph and virtual surgical planning platforms. The use of advanced digital analysis enhances diagnostic accuracy, facilitates surgical predictability, and improves communication with patients by providing clear visualizations of expected outcomes. These technologies also aid in achieving treatment precision, reducing operative time, and supporting postoperative stability.

The most striking skeletal change was the forward repositioning of the maxilla, as evidenced by the statistically significant increase in the SNA angle (*p* = 0.019). This aligns with findings by Marcelli et al. (2025), who similarly reported increases in SNA and SNB angles, along with a decrease in ANB—underscoring effective sagittal correction following orthognathic intervention [35].

Soft tissue responses in our patients were subtle but discernible—namely, mild increases in the nasolabial angle and improved facial convexity. These observations concur with the work of Lai et al. (2020), who found that Le Fort I maxillary advancement results in about 0.63 mm of soft tissue movement per 1 mm of bone shift in the anteromedial cheek region [32].

Our results regarding postoperative soft tissue adaptations are further supported by Nike et al. (2025), who documented measurable improvements in facial soft tissue symmetry over a 12-month period in Class II orthognathic patients, reinforcing the stability and aesthetic predictability of surgical-orthodontic treatment [36].

Our study used traditional 2D lateral cephalograms, but recent advancements advocate for multiplanar analysis. For instance, Perrotti et al. (2021) introduced the Total Face Approach (TFA)—a 3D cephalometric protocol that enables precise assessment of vertical skeletal discrepancies via CBCT superimposition, offering superior anatomical insight in orthognathic cases [37].

The minimal change observed in the SNB angle suggests that mandibular repositioning was limited or involved rotational adjustments rather than significant sagittal translation. This aligns with broader comparative evidence indicating that orthognathic surgery yields superior skeletal improvements, especially in jaw alignment, compared to orthodontic camouflage—where treatment effects are primarily dentoalveolar rather than skeletal. A systematic review by Alam et al. (2022) confirmed this, finding that orthognathic surgery achieves more pronounced skeletal corrections in adult Class III patients than camouflage approaches [38].

Our findings of profile enhancement—particularly in facial convexity—are consistent with Psomiadis et al. (2023), who demonstrated that combined surgical–orthodontic treatment yielded significantly greater perceived improvements (14–18%) in lower face aesthetics compared to camouflage treatment alone [39].

Lateral cephalograms, although extensively used for evaluating bone and soft tissue interactions, are fundamentally constrained by their two-dimensional projection, which restricts their capacity to depict intricate three-dimensional spatial alterations. Projection distortion, landmark overlap, and the inability to differentiate bilateral asymmetries or rotations may result in an underestimate of skeletal motions, especially in the vertical and transverse dimensions. A comparison analysis revealed substantial differences in several cephalometric values between two-dimensional radiographs and three-dimensional models, even with repeated tracing, suggesting that dependence on 2D imaging may obscure genuine three-dimensional inconsistencies [40].

Consequently, prudence is necessary while analyzing results concerning maxillary rotation, asymmetries, or vertical displacements.

The current results affirm that orthognathic surgery, especially maxillary advancement, yields consistent improvements in skeletal harmony and facial balance, whereas dental and soft tissue alterations, albeit more nuanced, are nevertheless clinically significant. These findings underscore the significance of integrating objective cephalometric studies with clinical acumen and patient-centered objectives. Recent comprehensive research underscores that digital planning and three-dimensional assessment significantly improve postoperative predictability and long-term stability in orthognathic surgery [41,42].

Moreover, the ongoing integration of artificial intelligence and virtual surgical planning is anticipated to augment diagnostic accuracy, treatment efficacy, and result consistency in orthognathic surgery [43].

Overall, the present findings confirm that orthognathic surgery, particularly maxillary advancement, provides reliable improvements in skeletal harmony and facial balance, while dental and soft tissue changes, though more subtle, remain clinically relevant. These results highlight the importance of combining objective cephalometric analysis with clinical judgment and patient-centered goals. Future research with larger cohorts, 3D imaging, and long-term follow-up will be essential to further validate these outcomes and refine interdisciplinary treatment protocols.

## 5. Study Limitations

This study provides valuable insight into skeletal, dental, and soft tissue changes following orthognathic surgery; however, several limitations must be acknowledged. The sample size was relatively small—25 patients from a single university center—which limits the generalizability of the findings. Expanding to a larger, multicenter cohort would allow for more robust statistical analyses and better representation of demographic and clinical diversity, including variations in sex, age, and skeletal pattern. The present sample size was enough to identify big clinical effects, but not medium or minor ones, according to the study’s power analysis. To validate these results and assess more nuanced changes associated with therapy, more study using bigger multicenter groups is required.

The observation period focused on short-term postoperative outcomes and did not evaluate long-term skeletal or soft-tissue stability. Longitudinal studies with follow-ups of two to five years would be necessary to assess relapse tendencies and adaptive responses over time.

Although digital cephalometric analysis with WebCeph ensured standardization, the use of two-dimensional imaging inherently restricts visualization of three-dimensional craniofacial structures. Minor landmark identification errors and radiographic quality variations may also introduce measurement bias. The integration of three-dimensional imaging methods such as cone-beam computed tomography (CBCT), 3D stereophotogrammetry, or digital facial scanning would provide a more comprehensive understanding of skeletal and soft-tissue dynamics.

Furthermore, this study evaluated only objective cephalometric parameters without incorporating patient-reported outcomes, such as satisfaction, quality of life, or psychosocial well-being. Including validated questionnaires and qualitative assessments in future work would yield a more complete understanding of postoperative impacts.

## 6. Future Perspectives

Future research should employ predictive modeling and machine-learning approaches to enhance preoperative planning and improve the predictability of surgical outcomes. Combining these computational methods with virtual surgical simulation and intraoperative navigation could further increase the precision and efficiency of orthognathic procedures.

Additionally, procedure-specific analyses should be performed by categorizing patients according to surgical technique (e.g., Le Fort I advancement, bilateral sagittal split osteotomy, genioplasty combinations) and baseline skeletal morphology (Class II, Class III, open bite, or asymmetries). Such stratification would clarify which treatment modalities yield the most stable and esthetic results across various clinical scenarios.

In summary, the present findings support the effectiveness of interdisciplinary, digitally guided orthognathic treatment. Continued research with larger sample sizes, advanced imaging technologies, extended follow-up periods, and inclusion of patient-centered outcomes will be essential to optimize future clinical protocols and enhance overall treatment success.

## 7. Conclusions

Orthognathic surgery, when combined with thorough orthodontic planning, enhanced skeletal balance and facial harmony in this group, with the most significant skeletal impact achieved by maxillary advancement (increase in SNA). Despite the moderate nature of soft tissue alterations, they exhibited a beneficial direction, and correlation studies validated the interrelationships among skeletal parameters (e.g., SNB–ANB) and between skeletal and dental variables (e.g., ANB–L1–NB°), indicating a coordinated response across tissues. These results emphasize the need for an interdisciplinary, data-driven approach that integrates standardized cephalometric analysis with virtual surgical planning. Digital instruments (e.g., AI-assisted landmarking, cephalometric dashboards, and 3D simulation) may augment diagnostic accuracy, better communication between surgeons and orthodontists, and refine patient counseling by establishing realistic expectations for both functional and aesthetic results. In practice, personalized planning that combines quantitative measurements with patient objectives should inform decision-making from presurgical decompensation to postoperative refining. Future investigations using 3D imaging techniques (CBCT, stereophotogrammetry) and extended follow-up are essential to confirm the permanence of skeletal correction and to more accurately assess soft-tissue adaptability. Stratified studies based on sex, age, and ethnicity, in addition to surgical patterns (e.g., Le Fort I advancement, BSSO, genioplasty combinations), will elucidate phenotype-specific responses. Ultimately, predictive modeling that integrates 3D morphometrics with machine-learning techniques may enhance case selection, refine soft-tissue predictions, and maximize multidisciplinary treatment strategies.

## Figures and Tables

**Table 1:** Patient characteristics and cephalometric measurements before and after orthognathic surgery.

Characteristic	Preoperative Median (IQR)	Postoperative Median (IQR)
Demographics		
Age (years)	28.0 (24.0, 33.0)	–
Sex, *n* (%)	Female: 11 (44%) Male: 14 (56%)	–
Skeletal parameters		
SNA (°)	83.6 (79.9, 84.9)	86.3 (80.4, 89.0)
SNB (°)	83.5 (76.8, 87.2)	84.0 (81.0, 86.0)
ANB (°)	0.9 (−4.9, 5.3)	3.0 (−0.2, 4.5)
Pogonion to N-Perpendicular FH (mm)	1.87 (−0.94, 2.31)	1.4 (0.4, 2.6)
Dental parameters		
U1–NA (°)	26 (21, 28)	22 (19, 29)
L1–NB (°)	24 (17, 27)	25 (19, 30)
Soft tissue parameters		
Nasolabial angle (°)	102 (92, 114)	105 (91, 117)
Facial convexity (°)	0 (−12, 7)	2 (−6, 5)

Values are presented as median (interquartile range, IQR) unless otherwise specified.

**Table 2:** Paired samples *t*-test comparing pre- and postoperative cephalometric measurements.

Variable	Mean Difference	SE Difference	t	df	*p*-Value
SNA (°)	−3.123	1.24	−2.511	24	**0.019**
SNB (°)	−1.147	1.41	−0.811	24	0.426
ANB (°)	−1.980	1.00	−1.977	24	0.060
Pogonion to N-Perpendicular FH (mm)	−1.571	1.21	−1.296	24	0.207
U1–NA (°)	0.465	1.87	0.249	24	0.806
L1–NB (°)	−2.968	1.55	−1.915	24	0.067
Nasolabial angle (°)	−6.950	8.83	−0.787	24	0.439
Facial convexity (°)	−0.502	4.12	−0.122	24	0.904

Student’s *t*-test for paired samples. Significant results (*p* < 0.05) are shown in bold.

**Table 3:** Correlation matrix of preoperative cephalometric, dental, and soft tissue variables in patients undergoing orthognathic surgery.

Variable	Age	SNA-PRE-OP	SNB	ANB	Pog to N-Perp FH	U1-NA°	L1-NB°	Nasolabial Angle	Facial Convexity
Age	—								
SNA-PRE-OP	0.016 (−0.382, 0.408), *p* = 0.941								
SNB	−0.080 (−0.460, 0.326), *p* = 0.705	0.473 (0.096, 0.732), *p* = 0.017							
ANB	0.099 (−0.308, 0.476), *p* = 0.637	0.058 (−0.345, 0.443), *p* = 0.784	−0.852 (−0.933, −0.689), *p* < 0.001						
Pog to N-Perp FH	−0.305 (−0.625, 0.102), *p* = 0.138	−0.066 (−0.450, 0.338), *p* = 0.753	0.463 (0.082, 0.725), *p* = 0.020	−0.563 (−0.784, −0.216), *p* = 0.003					
U1-NA°	−0.051 (−0.437, 0.351), *p* = 0.808	−0.064 (−0.448, 0.340), *p* = 0.762	0.382 (−0.015, 0.675), *p* = 0.059	−0.471 (−0.730, −0.093), *p* = 0.017	0.333 (−0.072, 0.643), *p* = 0.104				
L1-NB°	0.013 (−0.384, 0.406), *p* = 0.952	0.031 (−0.369, 0.421), *p* = 0.884	−0.559 (−0.781, −0.210), *p* = 0.004	0.652 (0.346, 0.833), *p* < 0.001	−0.244 (−0.583, 0.167), *p* = 0.240	−0.022 (−0.414, 0.376), *p* = 0.917			
Nasolabial angle	−0.084 (−0.464, 0.322), *p* = 0.691	0.325 (−0.081, 0.638), *p* = 0.113	−0.159 (−0.522, 0.252), *p* = 0.447	0.373 (−0.026, 0.669), *p* = 0.066	−0.056 (−0.441, 0.347), *p* = 0.791	−0.124 (−0.495, 0.285), *p* = 0.553	0.348 (−0.054, 0.653), *p* = 0.088		
Facial convexity	0.348 (−0.055, 0.653), *p* = 0.089	−0.048 (−0.435, 0.354), *p* = 0.819	−0.406 (−0.691, −0.013), *p* = 0.044	0.432 (0.045, 0.707), *p* = 0.031	−0.309 (−0.628, 0.098), *p* = 0.132	−0.230 (−0.573, 0.182), *p* = 0.268	0.419 (0.029, 0.699), *p* = 0.037	0.106 (−0.302, 0.481), *p* = 0.614	

**Table 4:** Correlation matrix of postoperative cephalometric, dental, and soft tissue variables in patients undergoing orthognathic surgery.

Variable	SNA-POST-OP	SNB After	ANB After	Pog to N-Perp FH After	U1-NA° After	L1-NB° After	Nasolabial Angle After	Facial Convexity After
SNA-POST-OP	—							
SNB After	0.410 (0.018, 0.693), *p* = 0.042							
ANB After	0.097 (−0.310, 0.474), *p* = 0.644	−0.459 (−0.723, −0.078), *p* = 0.021						
Pog to N-Perp FH After	0.133 (−0.276, 0.502), *p* = 0.525	0.153 (−0.258, 0.517), *p* = 0.465	−0.425 (−0.702, −0.036), *p* = 0.034					
U1-NA° After	−0.168 (−0.528, 0.243), *p* = 0.421	0.036 (−0.364, 0.425), *p* = 0.864	−0.435 (−0.709, −0.049), *p* = 0.030	0.440 (0.054, 0.711), *p* = 0.028				
L1-NB° After	−0.288 (−0.613, 0.121), *p* = 0.163	−0.157 (−0.520, 0.254), *p* = 0.454	0.340 (−0.064, 0.648), *p* = 0.097	0.421 (0.032, 0.700), *p* = 0.036	−0.696 (−0.856, −0.415), *p* < 0.001			
Nasolabial angle After	0.102 (−0.306, 0.478), *p* = 0.628	−0.225 (−0.569, 0.187), *p* = 0.280	0.435 (0.049, 0.709), *p* = 0.030	0.186 (−0.226, 0.541), *p* = 0.373	0.082 (−0.323, 0.463), *p* = 0.695	0.174 (−0.238, 0.532), *p* = 0.406		
Facial convexity After	0.108 (−0.300, 0.482), *p* = 0.609	−0.135 (−0.503, 0.275), *p* = 0.520	−0.327 (−0.639, 0.078), *p* = 0.111	0.329 (−0.076, 0.641), *p* = 0.109	−0.045 (−0.432, 0.357), *p* = 0.831	0.420 (0.029, 0.699), *p* = 0.037	0.053 (−0.350, 0.439), *p* = 0.802	

## Data Availability

All data regarding this manuscript can be requested from the corresponding author at andra.stancioiu@umft.ro and talpos.serban@umft.ro.
